# Prioritisation Assessment and Robust Predictive System for Medical Equipment: A Comprehensive Strategic Maintenance Management

**DOI:** 10.3389/fpubh.2021.782203

**Published:** 2021-11-17

**Authors:** Aizat Hilmi Zamzam, Ayman Khallel Ibrahim Al-Ani, Ahmad Khairi Abdul Wahab, Khin Wee Lai, Suresh Chandra Satapathy, Azira Khalil, Muhammad Mokhzaini Azizan, Khairunnisa Hasikin

**Affiliations:** ^1^Department of Biomedical Engineering, Faculty of Engineering, University of Malaya, Kuala Lumpur, Malaysia; ^2^Engineering Services Department, Ministry of Health Malaysia, Putrajaya, Malaysia; ^3^School of Computer Engineering, Kalinga Institute of Industrial Technology, Deemed to Be University, Bhubaneswar, India; ^4^Faculty of Science and Technology, Universiti Sains Islam Malaysia, Nilai, Malaysia; ^5^Department of Electrical and Electronic Engineering, Faculty of Engineering and Built Environment, Universiti Sains Islam Malaysia, Nilai, Malaysia

**Keywords:** medical devices, biomedical equipment, machine learning, prioritisation, prediction

## Abstract

The advancement of technology in medical equipment has significantly improved healthcare services. However, failures in upkeeping reliability, availability, and safety affect the healthcare services quality and significant impact can be observed in operations' expenses. The effective and comprehensive medical equipment assessment and monitoring throughout the maintenance phase of the asset life cycle can enhance the equipment reliability, availability, and safety. The study aims to develop the prioritisation assessment and predictive systems that measure the priority of medical equipment's preventive maintenance, corrective maintenance, and replacement programmes. The proposed predictive model is constructed by analysing features of 13,352 medical equipment used in public healthcare clinics in Malaysia. The proposed system comprises three stages: prioritisation analysis, model training, and predictive model development. In this study, we proposed 16 combinations of novel features to be used for prioritisation assessment and prediction of preventive maintenance, corrective maintenance, and replacement programme. The modified k-Means algorithm is proposed during the prioritisation analysis to automatically distinguish raw data into three main clusters of prioritisation assessment. Subsequently, these clusters are fed into and tested with six machine learning algorithms for the predictive prioritisation system. The best predictive models for medical equipment's preventive maintenance, corrective maintenance, and replacement programmes are selected among the tested machine learning algorithms. Findings indicate that the Support Vector Machine performs the best in preventive maintenance and replacement programme prioritisation predictive systems with the highest accuracy of 99.42 and 99.80%, respectively. Meanwhile, K-Nearest Neighbour yielded the highest accuracy in corrective maintenance prioritisation predictive systems with 98.93%. Based on the promising results, clinical engineers and healthcare providers can widely adopt the proposed prioritisation assessment and predictive systems in managing expenses, reporting, scheduling, materials, and workforce.

## Introduction

Medical equipment is one of the fundamental components contributing substantially to healthcare services effectiveness (1). The emerging and sophisticated equipment has significantly improved the community's health (2, 3). Procedures involved in healthcare services varying from diagnosis, therapeutics, rehabilitation, screening, prevention, and monitoring depend on medical equipment efficiency (4). The specialised equipment extensively assists healthcare practitioners during the early phase of symptom detection to curb health deterioration (5). Healthcare services delivery is almost impossible without proper maintenance of medical equipment (6). In addition, the devices need to be monitored for upkeeping performance in calibration, maintenance, restoration, training, and decommissioning, which are typically managed by clinical engineers (7). The clinical engineers in a healthcare facility are responsible for regulating and introducing an effective management programme for medical equipment reliability and safety (8). High technology innovation has elevated medical equipment complexity and eventually escalated the procurement and maintenance expenditures (6).

According to Kohani et al. (9), improving medical equipment in functionality depends on the internal electronic system. This dependency is vulnerable to electronic discharge that may cause unstable conditions and endanger users and patients. Hence, managing medical equipment maintenance is vital to ensure that the medical equipment operates according to the manufacturer's specification and guarantees patients and users' safety (10). Proper maintenance implementation can prevent possible failure or breakdown that affects the healthcare operations and may cause severe injury to the patients.

Kutor et al. (11) specifically reported that equipment failures are commonly due to inappropriate carriage and storage, preliminary breakdown, mishandling, lack of maintenance, environmental stress, random breakdown, improper restoration methods, and wear-out failure. The authors added that 50–80% of malfunctioning equipment is due to weak maintenance and a deficiency of highly skilled technicians. Furthermore, the authors highlighted that the four leading causes of those failures are preventable incidence, insufficient technical personnel, data deficiency, and lack of predictive maintenance. Therefore, medical equipment maintenance and management can be progressively improved by identifying the influential factors. According to the systematic review by Bahreini et al. (12), the authors summarised based on 29 previous studies that the management, resources, records, services, inspection, education, and quality control are among the affecting factors of medical equipment.

The World Health Organisation (WHO) divided the financial resources required for medical equipment maintenance into two categories: initial and operating expenditures (13). In addition, findings from Corciova et al. (14), indicated that 15–60% of the entire healthcare system operation were used in maintenance expenditures. Bahreini et al. (15) stated that unprofessional maintenance execution affected overall healthcare institutions' healthcare performance, safety, and expenses. Wu et al. (16) demonstrated that effective maintenance management managed to reduce operating costs by more than one million dollars and eventually enhanced equipment availability.

Several studies have been conducted to identify the global market value of medical equipment maintenance. The studies covered the preventive, corrective, and operational service types for several critical equipment with the involvement of the top manufacturers and service providers. As reported in MarketsandMarkets (17), the value of medical equipment maintenance for the global market was estimated at USD29 billion in 2018. The value is expected to grow to an estimated of USD48 billion by 2023. During this period, the estimated value of the annual growth rate over the time of investment, or Compound Annual Growth Rate (CAGR), will be 10.4%. FutureWise (18) estimated that this value exceeds USD62 billion with a CAGR of more than 10% for 2020–2027. Moreover, the projection indicates that the CAGR will grow by 9.4% from 2020 to 2030 (19). The key factors of these rising rates are the rising motivation for preventive maintenance, the demand for equipment, advanced financing mechanisms implementation, acquiring refurbished equipment, and a strict regulatory setting implementation.

Similar patterns can be observed in Malaysian healthcare facilities. The government invested approximately MYR27 million in public healthcare facilities by implementing new acquisitions and upgraded medical equipment programmes in 2018 (20). Additionally, the government executed a new leasing policy for six main medical equipment for a 5-year term beginning in 2019, including a servicing scheme for MYR19.7 million. One of the leading Malaysian private healthcare providers acquired medical equipment worth MYR136 million in 2019, a 32% increase compared to their previous year expenditure (21). These trends show that the substantial budget of medical equipment procurement and maintenance are imposed to deliver effective healthcare services.

The current medical equipment data availability in terms of equipment details, purchasing information, operational performance, and maintenance activities are critical in improving the equipment life cycle management. However, the appropriate technique is crucial in managing the big data that provides significant indicators in strategising maintenance management planning. This study addresses four identified gaps based on the literature review as follows:

- Lack of studies concentrated on comprehensive maintenance management, including preventive maintenance, corrective maintenance, and replacement programme.
- Inconsistency of mathematical approaches that require manual intervention in identifying the criteria weightages in reliability assessment.
- Inefficiency of the previous predictive models, which can be applied to the various types of medical equipment.
- None of the studies combines assessment and predictive models using the same unlabelled dataset of medical equipment.

Hence, this study aims to develop a predictive prioritisation model on the level of appropriate action to be taken by clinical engineers to ensure that medical equipment services in healthcare facilities are always prepared.

This study comprises the research background that presents the review of previous related studies and the motivation of proposing the models. Subsequently, the proposed methodology includes explanations on dataset, features, assessment techniques, predictive methods, machine learning algorithms, and performance evaluation. The results section demonstrates the assessment priority levels and the most accurate predictive model for each maintenance management. Next, the discussion section deliberates on the application of prioritisation assessment and predictive systems. The last section summarises the study findings, contributions, and recommendations.

## Literature Review

Numerous studies on the medical equipment assessment in healthcare facilities are presented in this section by identifying suitable keywords, specifically about medical equipment and devices. All factors in the study aim, equipment features, methodologies, techniques, expected output, and desired outcomes, were scrutinised thoroughly. Furthermore, the consideration of selected studies was based on real equipment database, quantitative methods assessment, and empirical studies.

Based on these criteria, 16 related studies were identified in assessing the medical equipment. The assessments were undertaken by analysing the medical equipment features comprising equipment particulars, equipment characteristics, and maintenance history records. In general, several techniques used in previous studies produced the indication of equipment reliability and strategised several appropriate maintenance activities. Besides, the study outcomes could improve the medical equipment reliability and availability by practising strategic maintenance management. Previous studies concluded that medical equipment assessment from several approaches might improve maintenance management implementation. This improvement may subsequently optimise the resources in terms of cost and workforce, strategise the best maintenance programme, and assist responsible parties in decision-making.

From these 16 studies, three (3) studies concentrated on improving preventive maintenance (22–24), three (3) studies aimed at strategising the best preventive or corrective maintenance (25–27), four (4) studies emphasised implementing the better replacement programme (28–31), and six (6) studies generated an indication of establishing the best maintenance strategy implementation (32–37). Several methods have been used to achieve the outcomes. Eleven (11) studies applied mathematical approaches (22, 25–31, 34–36), two (2) studies utilised Fuzzy Logic (23, 37), and one (1) study used Quality Function Deployment (24). Only two (2) studies applied supervised machine learning algorithms to process a 3-year database of equipment particulars, characteristics and maintenance records for infant incubators and defibrillators (32, 33). The findings of literature review are summarised as tabulated in Table 1.

**Table 1:** Summary of previous studies review.

**Authors (Region)**	**Maintenance Activities**			**Methods**	**Advantages and Disadvantages**
	**PM**	**CM**	**RP**		
Kovacevic et al. (32) (Bosnia and Herzegovina)	✓	✓		Supervised machine learning.	Advantage: High accuracy of predictive model. Disadvantage: Focused on one type of medical equipment (infant incubator).
Badnjevic et al. (33) (Bosnia and Herzegovina)	✓	✓		Supervised machine learning.	Advantage: High accuracy of predictive model. Disadvantage: Focused on one type of medical equipment (defibrillator).
Saleh et al. (24) (Italy)	✓			Quality function deployment.	Advantage: Effective preventive maintenance prioritisation. Disadvantage: Requirement of manual intervention for criteria weightages.
Hernandez-Lopez et al. (22) (Mexico)	✓			Mathematical model.	Advantage: Identification of equipment priority and preventive maintenance frequency. Disadvantage: Requirement of manual intervention for criteria weightages.
Jamshidi et al. (34) (Canada)	✓	✓		Fuzzy failure modes and effect analysis.	Advantage: Maintenance strategy through medical equipment prioritisation. Disadvantage: Requirement of manual intervention for criteria weightages.
Faisal et al. (28) (Egypt)			✓	Analytical hierarchy process (AHP).	Advantage: Medical equipment replacement prioritisation. Disadvantage: Requirement of manual intervention for weight grade scores for each equipment type.
Tawfik et al. (37) (Egypt)	✓	✓		Fuzzy logic.	Advantage: Cost optimisation and prioritisation of various types of medical equipment. Disadvantage: Focused on preventive maintenance and corrective maintenance.
Jarikji et al. (30) (Lebanon)			✓	Mathematical model.	Advantage: Replacement prioritisation based on lifespan of medical equipment. Disadvantage: Requirement of manual intervention for criteria weightages for limited types of equipment.
Aridi et al. (31) (Lebanon)			✓	Multi-criteria decision making (MCDM).	Advantage: Replacement prioritisation based on actual usage for various types of medical equipment. Disadvantage: Requirement of manual intervention for criteria weightages
Hamdi et al. (27) (Jordan)	✓	✓		Mathematical model.	Advantage: Maintenance prioritisation and proper scheduling based on patient safety and healthcare quality sensitivity. Disadvantage: Requirement of manual intervention for criteria weightages.
Hutagalung et al. (36) (Indonesia)	✓	✓		Analytical hierarchy process (AHP).	Advantage: Prioritisation of preventive maintenance and corrective maintenance based on equipment ranking. Disadvantage: Requirement of manual intervention for criteria weightages.
Taghipour et al. (25) (Canada)	✓	✓		Analytical hierarchy process (AHP).	Advantage: Prioritisation of maintenance based on equipment criticality. Disadvantage: Requirement of manual intervention for criteria and sub-criteria weightages.
Ben Houria et al. (26) (Tunisia)	✓	✓		Analytical hierarchy process (AHP), technique for order performance by similarity to ideal solution (TOPSIS), and mixed-integer linear programming (MILP).	Advantage: Prioritisation of maintenance based on risk level for various types of medical equipment. Disadvantage: Requirement of manual intervention for criteria and sub-criteria weighting values.
Oshiyama et al. (29) (Brazil)			✓	ABC analysis and paraconsistent annotated logic (PAL) analysis.	Advantage: Replacement prioritisation based on corrective maintenance record for various types of medical equipment. Disadvantage: Determination of factors involved in the proposed method was based on assumption. The inconsistency was detected during the classification process.
Saleh and Balestra (23) (Italy)	✓			Quality function deployment and fuzzy logic.	Advantage: Preventive maintenance prioritisation based on the most important six criteria for various types of medical equipment. Disadvantage: Requirement of manual intervention for preventive maintenance criteria weightages.
Ismail et al. (35) (Lebanon)	✓	✓		Failure modes and effect analysis and monte carlo simulation.	Advantage: Risk prediction for maintenance prioritisation. Disadvantage: Decision for preventive maintenance or corrective maintenance based on technical personnel discretion.

*PM, Preventive Maintenance; CM, Corrective Maintenance; RP, Replacement Plan*.

A review of previous studies led to the identification of several gaps. First, none of the studies contributed and concentrated on comprehensive strategic maintenance management, including preventive maintenance, corrective maintenance, and replacement programme. The reported studies only focused on corrective maintenance, preventive maintenance, or replacement plan, which provided only preliminary analyses to the healthcare facility providers. Secondly, the mathematical approaches used in previous studies comprised a manual intervention that required criteria weightages determined by the clinical engineers. The values may vary depending on their knowledge, and the approaches may generate inconsistent results. Besides, the clinical engineers might overlook other essential features required for strategic maintenance management. Therefore, Kovacevic et al. (32) and Badnjevic et al. (33) utilised the machine learning technique in predicting infant incubators and defibrillators. However, both studies only focused on one type of equipment. The studies provided an insufficient framework to be applied in the healthcare service setting where myriad equipment, either critical or non-critical devices, are involved. The literatures also discovered that none of the studies provided the combination techniques of medical equipment assessment and priority prediction by measuring the same features and criteria on the unlabelled dataset. A comprehensive analysis is needed to prioritise the best decision-making strategies in providing optimum healthcare service.

The proposed model contributes to the comprehensive strategic maintenance management at the end of the study, covering preventive maintenance, corrective maintenance, and replacement programme. The proposed model shows that machine learning techniques successfully overcome the users' manual intervention in identifying the criteria weightages. Hence, the proposed system outcomes enable a rapid and optimised decision on strategic maintenance management practise by measuring and observing the data pattern based on the medical equipment record. The various medical equipment datasets in Malaysian public healthcare facilities were used to measure every development stage in this study and not strictly on a specific type of medical equipment. Therefore, the prioritisation assessment and predictive systems will be more robust to any type of medical equipment in any healthcare facility.

In addition, the proposed model will aid the clinical engineers in prioritising the strategic maintenance management that covers preventive maintenance, corrective maintenance, and replacement programme. The model assesses and prioritise the existing equipment and predict the prioritisation of new equipment. Furthermore, the predictive model will improve outage scheduling, operational stability, equipment reliability, and effective spare part management.

## Materials and Methods

The specified features and various kinds of medical equipment were analysed for medical equipment assessment in this study. The proposed model consists of three stages. The first stage comprises the development of a medical equipment prioritisation assessment system. The second stage consists of the classification model training of the predictive prioritisation system, while the third stage establishes the development of a comprehensive strategic maintenance management prioritisation predictive system. Unlike previous studies, this study measured the collective set of various medical equipment datasets features to assess and predict the medical equipment state.

**Figure 1:**
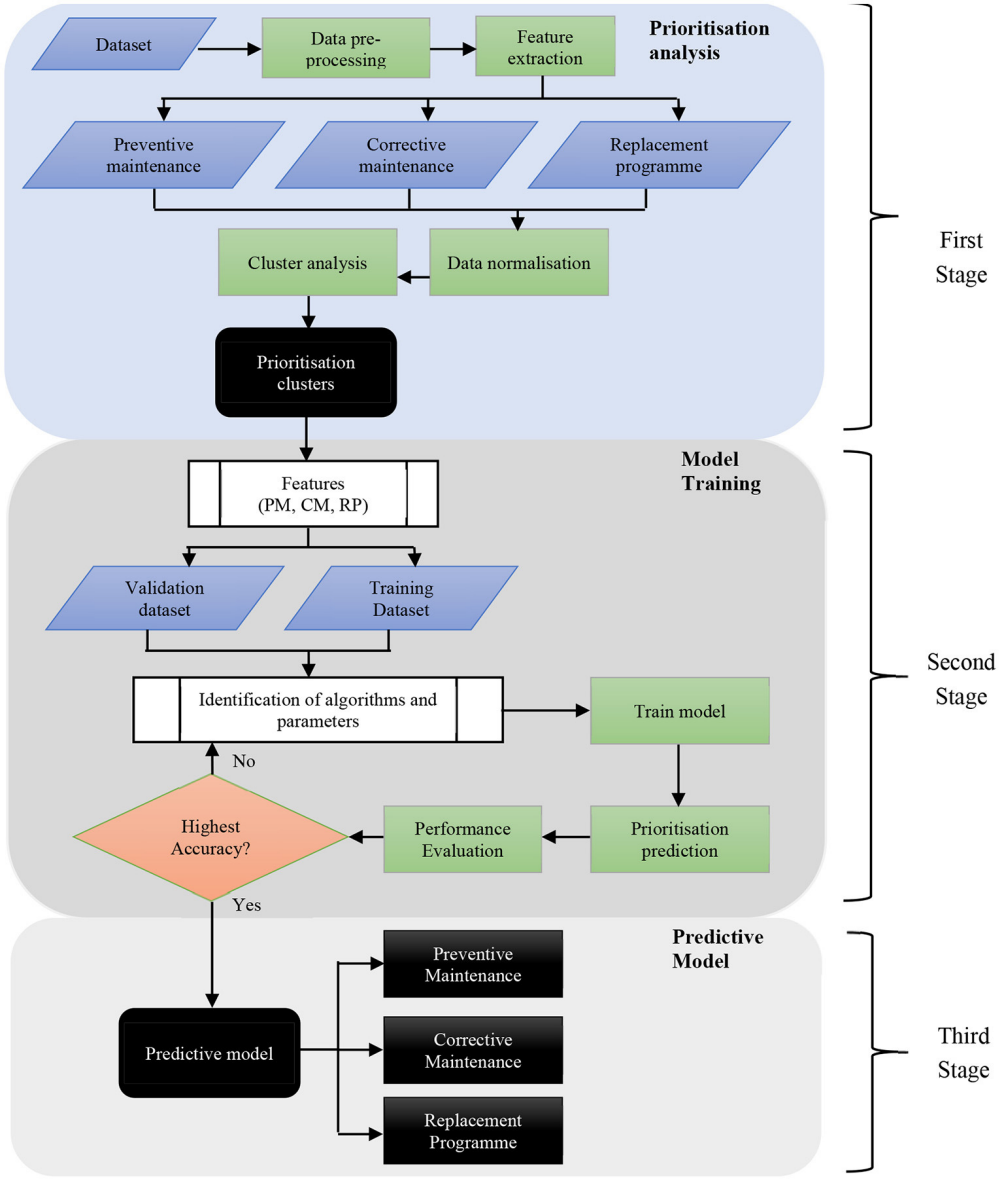
The block diagram of the prioritisation assessment and medical equipment development prediction systems.

Figure 1 exhibits the prioritisation assessment system and predictive prioritisation system proposed model. The proposed model was developed to predict the prioritisation of three primary strategic maintenance management, namely preventive maintenance, corrective maintenance, and replacement programme. Sixteen medical equipment features were extracted from the datasets consisting of 13,352 medical equipment and were automatically clustered using the unsupervised k-Means algorithm (38–42). Three priority numbers for each preventive maintenance, corrective maintenance, and replacement programme were determined from the 16 medical equipment features. Subsequently, the outputs were fed into the second stage, which is model training. Six different machine learning algorithms were present in this stage: (1) Decision Tree, (2) Linear Discriminant, (3) Naïve Bayes, (4) Support Vector Machine, (5) K-Nearest Neighbour, and (6) Bagged Trees were tested by measuring the specified features of various types of medical equipment. The reason of selecting these six classifiers were because of its suitability with the characteristics of medical equipment dataset involved in this study. The Decision Tree, Bagged Trees, K-Nearest Neighbour, Support Vector Machine, and Naïve Bayes techniques were observed to be highly accurate predictive models performed in similar previous studies (32, 33). Another classifier namely Linear Discriminant was also applied because this technique is good for multiclass and broad datasets (43). Moreover, all the proposed classifiers are capable of measuring the numerical predictors in the dataset.

The dataset was divided into two for training and validation processes. The performance results of training and validation were evaluated to measure the prediction accuracy. The algorithm that produced the highest performance evaluation result was selected as the predictive prioritisation model in the third stage for effective medical equipment maintenance management that covers all three strategic maintenance management.

### Medical Equipment Dataset

In developing medical equipment prioritisation assessment and predictive prioritisation systems, 13,352 medical equipment dataset samples were extracted. The medical equipment dataset was obtained from the asset management record from 2015 to 2020 from 19 medical equipment categories. These 19 medical equipment categories were located at public health clinic facilities in 10 states in Malaysia. The medical equipment categories and unit numbers are tabulated in Table 2.

**Table 2:** Medical equipment category and number.

**Medical equipment**	**Quantity**
Chemistry analyser fully automated	137
Bilirubinometers, Lab	777
Automatic defibrillator	861
Manual defibrillators	204
Densitometers	46
Incubators, Infant	31
Infusion pumps, General-Purpose	16
Laryngoscopes	1,473
Physiologic monitoring systems	1,251
Nebulisers, Non-heated	2,297
Pulse oximeter	1,319
Phototherapy units	28
Radiographic/Fluoroscopic systems, general-purpose	151
Manual pulmonary resuscitators	833
Pharmacy weighing machine	690
Ultrasonic scanner	647
Sensitometers, Radiographic	44
Autoclave unit	2,417
Treadmills	130

There were 16 medical equipment features used as the inputs of the prioritisation assessment system in the first stage. The identification of these features was referred to the review of the previous 16 studies by using thematic analysis as presented in (44). The features were determined by identifying the common themes extracted from the previous studies to ensure that important aspects of the medical equipment were considered. From the results, there were 8 categories of inputs to assess the medical equipment reliability. Therefore, the inclusion of 16 features was correlated with eight categories of assessment inputs. Moreover, the proposed features were taken into consideration by observing all the important aspects in developing a comprehensive model. Furthermore, the proposed features were also associated with related Malaysian Standard (45). Therefore, by applying the proposed combination of features, the accurate and precise assessment, and predictive model can be developed and subsequently, in line with the national standard.

The segregation of the features based on preventive maintenance, corrective maintenance, and replacement programme was also determined from the review. Thus, a standard form of features was applied in preventive maintenance, corrective maintenance, and replacement programme systems development. Each feature consisted of several criteria based on the samples of medical equipment characteristics. The criteria are numerical and used for clustering processes in the first stage. The appropriate combination of features may assist the machine learning application in finding the requirement of the descriptions for generating the desired output (46). The novel features and criteria of the medical equipment sample are tabulated in Table 3. Each medical equipment sample was categorised accordingly based on the current characteristics specified in the asset management record. The clinical engineers frequently updated the medical equipment status after the upkeeping works were completed. The combination of these features was never tested before for comprehensive strategic maintenance management.

**Table 3:** Medical equipment features and criteria.

**Features**	**Criteria**
Equipment age	Age number (vary)
Support service	Obsolescence (1) Available (0)
Asset condition	Beyond economical repair (BER) (2) Proposed for disposal (1) Active (0)
Function	Life support (5) Therapeutic (4) Diagnostic (3) Analytical (2) Miscellaneous (1)
Preventive Maintenance Status	Not in the schedule (2) Open (1) Completed (0)
Number of missed Planned Preventive Maintenance (PPM)	Number of missed PPM (vary)
Request to repair time (Response time)	Duration of technical personnel to respond on the failure equipment (average day)
Maintenance requirement	PPM (Twice annually) and Statutory Certification (5) PPM (Twice annually) (4) PPM (Once annually) and Calibration (3) PPM (Once annually) (2) Routine Inspection (1)
Maintenance complexity	Extensive maintenance (3) Average maintenance (2) Visual inspection and basic cheque (1)
Repair time	Mean time to repair (average day)
Downtime	Duration of equipment malfunction (average/year)
Number of failures	Number of failures on the equipment (vary)
Asset status	Functioning (0) Malfunctioning (1)
Backup or alternative unit	Yes (0) No (1)
Operations	Criticality (6–1)
Maintenance cost	The accumulative cost of repair work (vary)

*PPM, Planned Preventive Maintenance; BER, Beyond Economic Repair*.

*(5)—Ranges of rating*.

#### Equipment Age

The equipment life span depends on the equipment age. Therefore, this parameter was calculated by subtracting the equipment purchase date from the cut-off date, which is set to be October 2020 in this study.

#### Support Service

High technological aspects are adopted in manufacturing medical equipment. The requirement of regular maintenance and replacement of consumable parts shall be performed and carried out by the authorised parties to ensure the equipment can function at the optimum level. The obsolescence of the spare parts and service providers' availability will jeopardise the medical equipment maintenance activity. This study's obsolescence status was determined by considering the equipment's manufacturer's equipment expected life cycle.

#### Asset Condition

The current three medical equipment conditions are active, proposed for disposal, and beyond economic repair (BER) (45). The equipment is considered active if it can operate at an optimum level according to the manufacturer specifications. Some equipment is still functioning but proposed for disposal due to unnecessary or no longer being used. The proposal is to reserve limited operational space or reduce any costs imposed on the maintenance works. The unit can be declared as BER whenever the equipment is malfunctioning. The repair cost exceeds the specific percentages of equipment value or any reasons specified by the healthcare facility administrator.

#### Function

The term function refers to the main purpose or service intention of the medical equipment. Five criteria are involved given the function factor, namely life support, therapeutic, diagnostic, analytical, and miscellaneous. The equipment is classified under life support if the unit failure cause harm to the life or death. Therapeutic equipment refers to the units treating or providing a remedy for any illness or disease experienced by the patient. The medical equipment used to detect any illness or disease is categorised under diagnostic equipment. The analytical equipment involves the laboratory procedure of analysing patients' samples, whereas miscellaneous equipment refers to any units used to support the primary healthcare and medical activities.

#### Preventive Maintenance Status

The preventive maintenance information is an essential factor in determining the medical equipment condition. This factor consists of three criteria in this study: “completed,” “open”, and “not in schedule.” The “completed” term refers to planned preventive maintenance (PPM) activities undertaken successfully per the manufacturer's recommendations in the manual book. Any annual PPM of medical equipment scheduled for the current year but not performed is considered “open” maintenance work. This indication is important to alert the clinical engineers to initiate the PPM activity. The term “not in schedule” denotes that the clinical engineers are not preparing the PPM schedule for the equipment under their supervision. This situation may lead to incomplete PPM for the current year.

#### The Number of Missed Planned Preventive Maintenance

As explained above, the status “not in schedule” may lead to the incompleteness of PPM for the current year or the first frequency of PPM for the equipment that requires PPM twice annually. The possibility of PPM being skipped for the previous year might occur if appropriate actions are not properly taken. The more frequently PPM is missed will cause increased chances of medical equipment failure.

#### Request to Repair Time

Request to Repair Time or Response Time refers to the duration starting from the failure report launched by the user until the technical personnel attend for identification work of failure. The longer the Response Time, the longer medical equipment is unable for use, the failure cause remains unknown, and eventually causes the interruption of healthcare services to the patient.

#### Maintenance Requirement

Maintenance requirement refers to the maintenance work performed by a competent person per the manufacturer specifications, national authorised bodies, and the healthcare facility administrator. Several equipment manufacturers set a specific maintenance interval annually. Statutory certification is required for certain equipment such as radiographic equipment that exposes radiation within the surrounding areas (47). This equipment shall be inspected, and the radiation exposed shall be controlled within the specified limits (48, 49). The calibration of specific measuring medical equipment is required to generate an accurate result. Conversely, “Routine Inspection” refers to maintenance activities that involve physical cheques, regular operational tests, and other relevant qualitative tests.

#### Maintenance Complexity

Maintenance complexity refers to the level of difficulties in performing the maintenance procedures. This feature consists of three criteria, which are extensive maintenance, average maintenance, and basic inspection. Extensive maintenance refers to the complex system where the medical equipment is equipped with mechanical systems such as pneumatic, hydraulic, motorised, and others. According to Fenningkoh and Smith (50), the equipment comprises of the complex systems involves the most substantial maintenance. Therefore, well-trained, certified, and highly skilled workers in needed to carry out this activity ensuring the overall system runs accordingly to the manufacturer's specifications and statutory requirement. Furthermore, it requires specific tools, and the execution of maintenance procedures is time consuming. The average maintenance requires several cheques and testing, such as performance and safety tests. Basic inspection involves visual examination, operational tests, battery replacement, and cleaning.

#### Repair Time

Repair Time or Mean Time to Repair (MTTR) is the duration is taken by technical personnel to perform repair, restore, and rectification work. The more time is taken by technical personnel to rectify the equipment, the longer the interruption of healthcare services.

#### Downtime

Downtime refers to the duration the medical equipment is under failure conditions and cannot operate by the manufacturer specifications throughout its useful life. Downtime is measured by subtracting the failure report time launched by the user from the repair work completion time. The unit of equipment downtime is the average downtime per year. The longer the equipment downtime, the longer the equipment cannot be safely used and the longer the patient's medical service is interrupted. The downtime also indicates the overall performance of medical equipment in terms of functionality.

#### The Number of Failures

The number of failures refers to the frequency of events the equipment is unable to be utilised during its useful life. The failures can be determined by observing the failure report launched by the users. Attention must be given to the equipment that frequently fails in operation. Excessive failures indicate that the equipment must be observed frequently and inspected thoroughly to prevent further damage to the system.

#### Asset Status

Preventing medical equipment failure is crucial to increase machine reliability. The asset status consists of only two criteria: functioning and malfunctioning. The medical equipment that can operate following manufacturer specifications without receiving any failure report from the user is categorised as functioning. Nevertheless, the equipment is categorised as malfunctioning when the medical equipment is unable to operate accordingly. Simultaneously, the user has launched a breakdown report when the unit fails to provide medical services to the patients.

#### Backup or Alternative Units

The backup and alternative unit refer to any substitute equipment used temporarily to provide medical services if the main equipment malfunctions. Due to the criticality and substantial factors in providing vital medical services, some alternative or backup units that can temporarily be used to avoid service interruption are available. The backup units ensure that the users can continue their duties, and the breakdown equipment maintenance can be performed immediately.

#### Operations

Operations refer to the utilisation rate, which indicates the equipment usage in providing medical services in healthcare facilities. The indication of utilisation rate is according to the average operating hours of the medical equipment to the healthcare facility working hours per day. In this study, the feature is divided into six criteria, where indicates the average of equipment operating hours per day as follows:

- (1) - < 2 h
- (2) - 2 h ≤ × < 3.5 h
- (3) - 3.5 h ≤ × < 5 h
- (4) - 5 h ≤ × < 6.5 h
- (5) - 6.5 h ≤ × < 8 h
- (6) - ≥ 8 h

The segregation is made through continuous monitoring by maintenance personnel and based on the utilisation degree by users. These criteria were already registered in the asset management record system.

#### Maintenance Cost

This feature refers to the total cost imposed to rectify the malfunctioned equipment. The repair cost involves the purchasing of materials, labour, and other related expenses.

### Medical Equipment Prioritisation Assessment System

In the prioritisation analysis stage, we proposed three groups which are denoted as high, medium, and low. The segregation into three levels was proposed based on the operation and implementation of further actions in adequate stages, appropriate to urgency, criticality, and seriousness. These group numbers were applied to all three strategic maintenance management, namely preventive maintenance, corrective maintenance, and replacement programme. Each proposed feature as discussed above, was labelled using the k-Means clustering technique for various types of medical equipment. Hence, the priority of each strategic maintenance management can be recognised.

The values significantly varied and were dynamic as the features and criteria were different among others. Therefore, data normalisation was required to ensure that the distance of each value of features was weighted equally. Otherwise, the large values of each equipment feature can dominate the entire measurement and create an outlier. Therefore, the data normalisation of the features in this study was scaled with the mean being equal to zero (0), and the standard deviation is equal to one (1). The following equations were used to calculate the normalisation process:


$$\begin{array}{r} \text{z}-\text{score}=\frac{\chi -\mu }{\sigma }\\ \sigma =\sqrt{\frac{1}{\text{n}-1}\overset{\text{n}}{\underset{\text{i}=1}{\sum }}\left(\chi \text{i}-\text{ X}\right)} \end{array}$$

where,

χ = Data

μ = Mean

σ = Standard deviation

n = Number of equipment.

The dataset was clustered into three groups by using the k-Means clustering technique after the execution of normalisation. Two parameters of k-Means were proposed prior to the clustering processes which are, distance metric and replicate. The distance metric was measured by calculating the similarity between two observations (51). The Squared Euclidean distance in this study was used based on the following equation:


$$\begin{array}{l} \text{d}\left(\text{x},\text{c}\right)=\left(\text{x }-\text{ c}\right){\left(\text{x }-\text{ c}\right)}^{\prime } \end{array}$$

where,

*d* = Distance

*x* = Equipment feature value

*c* = Centroid (The mean of the points in the cluster)

The replicate option is a function where different starting centroids are executed numerous times according to the specified number before choosing the minimum sum of the distances between the observations and centroids (52). Therefore, both setups were applied to all three assessment systems of strategic maintenance management prioritisation before analysing the clusters. Thus, the outputs of the medical equipment prioritisation assessment level after the cluster analysis process consist of three priority levels that apply to all strategic maintenance management.

The priority of preventive maintenance requires nine features of the medical equipment dataset as tabulated in Table 4. The nine features of the medical equipment dataset were used to cluster the equipment into three groups that refer to high, medium, and low priority. The number of equipment units involved was 13,352. Meanwhile, the prioritisation of corrective maintenance involves nine features of the medical equipment dataset. Similar to preventive maintenance, nine features of the medical equipment was used to cluster the equipment into three group of priorities. However, only malfunctioning equipment in the asset status criteria was taken in this clustering process. Hence, only 1,028 equipment units were chosen. Next, the priority of the replacement programme requires 11 features of the medical equipment dataset. These features were used for the priority clustering process. The size of the equipment involved is 13,352. The summary of medical equipment features for all three strategic maintenance management is presented in Table 4. From the table, three features were applied to all three strategic maintenance management, namely function, operations, and the number of failures.

**Table 4:** Features of medical equipment.

**Preventive maintenance**	**Corrective maintenance**	**Replacement programme**
Age	Function	Age
Function	Response time	Obsolescence
Preventive maintenance status	Maintenance complexity	Function
Missed PPM	Repair time	Maintenance requirement
Maintenance requirement	Number of failures	Downtime
Maintenance complexity	Backup and alternative unit	Number of failures
Downtime	Operations	Asset status
Operations	Maintenance cost	Backup and alternative unit
Number of failures	Asset status	Operations
		Maintenance cost
		Asset condition

*PPM, Planned Preventive Maintenance*.

### Prioritisation Predictive System

The prioritisation predictive system development involved two stages illustrated in Figure 1, namely model training and predictive model. The predictive prioritisation system predicts the medical equipment strategic maintenance management priority level that covers preventive maintenance, corrective maintenance, and replacement programme. The input data were labelled with priority levels during the prioritisation assessment system process.

**Figure 2:**
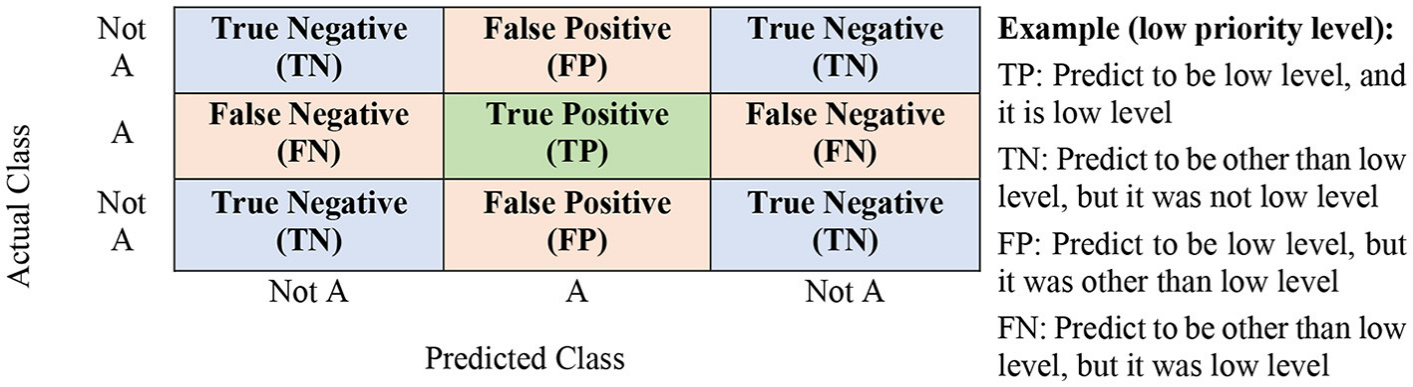
Confusion matrix.

The processes of all model training were executed for all strategic maintenance management by considering all related features. The labelled data for each strategic maintenance management was divided into a subset of datasets for training and validation purposes to evaluate the performance of each classifiers' models and protect against overfitting. Therefore, the cross-validation value was set to 10 (10)-folds. The outputs of models' training by considering all parameters were recorded. Six classifier algorithms were used to train the models in developing a predictive prioritisation system, and the predicted outcomes generated from all classifiers were observed. Before executing the models' training by applying the labelled datasets of every strategic maintenance management, all six parameters of classifiers were configured as tabulated in Table 5. The performance evaluation was undertaken on all predicted outcomes generated for all strategic maintenance management after the training of the models is completed. Figure 2 illustrates the confusion matrix and its components.

**Table 5:** Classification algorithm parameters.

**Algorithm**	**Parameters**	
Decision tree	Split criterion Maximum splits number	Gini's diversity index 100
Linear discriminant	Pre-set Covariance structure	Linear Full
Naïve bayes	Distribution name for numerical predictors Kernel type Support	Kernel Gaussian Unbounded
Support vector machine	Kernel function Scale Box constraint level Multiclass method Standardise data	Cubic Automatic 1 One-vs-One True
K-Nearest neighbour	Pre-set Neighbour number Distance metric Distance weight Standardise data	Fine 1 Euclidean Equal True
Bagged trees	Ensemble method Learner type Maximum splits number Number of learners	Bag Decision tree 13,351 30

The performance evaluation that consists of classification models' accuracy, precision, recall, and f-score can be determined from the confusion matrix (53, 54). The following formulas can describe the accuracy, precision, recall, and f-score:


$$\begin{array}{r} \text{Accuracy}=\frac{\text{TP}+\text{TN}}{\text{TP}+\text{FP}+\text{TN}+\text{FN}}\\ \text{Precision}=\frac{\text{TP}}{\text{TP}+\text{FP}}\\ \text{Recall }\left(\text{Sensivity}\right)=\frac{\text{TP}}{\text{TP}+\text{FN}}\\ \text{F Score}=\frac{\text{Precision }\left(\text{avg}\right)\times \text{Recall}\left(\text{avg}\right)}{\text{Precision }\left(\text{avg}\right)+\text{Recall}\left(\text{avg}\right)} \end{array}$$

The performance evaluation results generated from the six classifiers for all three strategic maintenance management were recorded, compared, and verified.

The third or final stage in developing the predictive prioritisation system is establishing the highest performance measurement in accuracy, precision, recall, and f-score for preventive maintenance, corrective maintenance, and replacement programme. The comprehensive strategic maintenance management can be achieved by establishing the highest performance measurement produced by the classification algorithm.

## Results

The prioritisation system results in the first stage and the predictive prioritisation system in the second stage are presented in this section. The priority level, which is the prioritisation system output, will input the prioritisation predictive system to generate the predicted output. Therefore, the dependency of both systems is crucial in producing the final outcomes.

### Prioritisation Assessment System

#### Preventive Maintenance

The preventive maintenance prioritisation system was developed using the proposed nine medical equipment features. In the prioritisation analysis stage, 13,352 medical equipment from 19 equipment categories were divided into three priority levels: high, medium, and low, as tabulated in Table 6.

**Table 6:** Medical equipment priority levels.

	**Cluster priority**								
	**Preventive maintenance**			**Corrective maintenance**			**Replacement programme**		
	**High**	**Medium**	**Low**	**High**	**Medium**	**Low**	**High**	**Medium**	**Low**
Number of medical equipment	3,603	3,107	6,642	375	651	2	4,351	1,027	7,974

The cluster analysis for the preventive maintenance prioritisation system is presented in Table 7. The analysis demonstrated that the equipment characteristics reflect the priority level segregation. Seventy-four percentage of the total equipment aged below 10 years and completed preventive maintenance based on the schedule were considered as low priority. In addition, 88% of the total equipment have less or equal to only one time of missed PPM is also considered as low priority. Furthermore, in this cluster, most equipment requires only one PPM or routine inspection per annum and low complexity of the technical maintenance work. The low priority cluster, consisted of 73% of the total equipment with no downtime and <11 failures.

**Table 7:** Findings of preventive maintenance prioritisation system.

**Feature**	**Range**		
	**Low**	**Medium**	**High**
Age	• 0–41 years • 73% is <10 years.	• 0–28 years • 69% between 2 and 15 years	• 0–40 years; • 70% more than 10 years
Preventive maintenance status	Completed	Incomplete and not on the schedule	Incomplete and not on the schedule
Missed PPM	• 0–5 times • 57% none • 31% is 1 time • 12% is more than 1 time.	• 0–4 times • 70% none • 30% in between 1 and 4 times.	• 0–7 times • 78% in between 1 and 7 times
Maintenance requirement	1–2 (1 × PPM frequency and routine inspection)	2–4 (2 × PPM, calibration, and 1 × PPM)	5 (2 × PPM and statutory certification)
Maintenance complexity	1 (Visual inspection and basic cheque)	2 (Average maintenance)	3 (Extensive maintenance)
Downtime	• 0–252 average days • 73% is none • 9% is within a day • 18% more than 1 day.	• 0–242 average days • 62% is none • 13% is within a day • 25% more than 1 day.	• 0–548 average days • 13% is none • 12% is within a day • 75% is more than 1 day.
Number of Failures	• 0–11 times • 73% is none • 27% between 1 and 11 times.	• 0–14 times • 62% is none • 38% between 1 and 14 times.	• 0–41 times • 13% is none • 84% between 1 and 14 times • 2.5% more than 15 times.
Function (type)	• 35% (1, 2) • 19% (3) • 46% (4, 5).	• 16% (1, 2) • 5% (3) • 79% (4, 5).	• 77% (1, 2) • 19% (3) • 4% (4, 5).

*PPM, Planned Preventive Maintenance*.

*The percentages stated in the table reflect to the unit of equipment*.

The observation of the medium priority cluster showed that the highest equipment age is 28 years in which, 69% of total equipment aged in between 2 and 15 years in operations. Furthermore, the medium priority also consisted of equipment with “incomplete” and “not in schedule” categories of preventive maintenance. They required a maximum of two frequency PPM per annum. The highest recorded number of missed PPM is four (4) times, in which 30% of total equipment were having missed PPM between 1 and 4 times. The medium priority also consisted of 62% of total equipment were 100% available, in which the highest downtime with 242 days per annum and 62%, whereas the highest number of failures was 14. In contrast, the high priority equipment consisted of equipment age up to 40 years in service, where 70% of them are more than 10 years. Besides, the high priority also consisted of equipment with uncompleted work, and no assignment of PPM schedule. Seventy-eight percentage of equipment missed the PPM in between 1 and 7 times, requires twice of PPM annually, and complies with the statutory certification regulated by the authority body and needs maintenance with extensive procedures. In addition, the equipment failure up to 41 times could lead to the highest downtime with an average of 547 days.

The analysis tabulated in Table 7 demonstrates that the k-Means clustering technique with nine proposed features from various categories of medical equipment successfully clustered 13,352 units of medical equipment. The medical equipment with high characteristics regarding age, preventive maintenance status, uncompleted PPM, maintenance requirement, maintenance complexity, downtime, number of failures, and operations are the majority in the high cluster. Hence, the clustering technique developed a practical preventive maintenance prioritisation system for medical equipment by applying the Squared Euclidean distance in k-Means. The cluster analysis in the first stage was executed several times by selecting the Squared Euclidean for the distance metric and the replicating number was proposed to be set as five (5) to get the best centroid points. These centroid points are essential to split the medical equipment samples to the appropriate priority level region based on the specified number of clusters and generate better clustering segregation of medical equipment priority levels.

The analysis also discloses that the proposed clustering technique measures the dataset of medical equipment without giving a priority specifically to the function or type of equipment. Other eight criteria also contributed significantly in assessing the medical equipment for preventive maintenance prioritisation. The prioritisation for preventive maintenance is assigned by measuring the pattern of the nine features in the dataset, and locating the nearest Euclidean distance with the centroids. In other words, the possibility of critical devices based on functionality or type can be assigned to the low priority of preventive maintenance if the equipment still new, maintained properly, and able to operate well.

#### Corrective Maintenance

The corrective maintenance prioritisation system was developed based on the proposed eight medical equipment features of the malfunctioning medical equipment. The output of this system was divided into three priority levels, and the quantities of medical equipment at each level are as tabulated in Table 6. The findings of the clustering technique on medical equipment for corrective maintenance prioritisation systems are presented in Table 8. The analysis of corrective maintenance priority considered only medical equipment breakdown. Thus, the asset status of all priorities is malfunctioning. The low priority proved that the highest reading of response time is six (6) days, the repair time is 29 days, while the failure frequency is eight (8) times. Besides, a backup or alternative unit is available if the equipment breakdown and the highest maintenance cost is up to MYR8,000.

**Table 8:** Findings of the corrective maintenance prioritisation system.

**Feature**	**Range**		
	**Low**	**Medium**	**High**
Asset status	Malfunctioning	Malfunctioning	Malfunctioning
Response time	Less than 6 average days	• 0–69 average days • 31% is none • 54% is within a day • 15% is more than a day.	• 0–148 average days • 20% is none • 41% is within a day • 29% is more than a day.
Maintenance complexity	Extensive maintenance	Basic cheque and average maintenance-−80.5%	Extensive maintenance-−94.4%
Repair time	• Less than 29 average days	• 0–253 average days • 31% is none • 17% is within a day • 51% is more than a day.	• 0–478 average days • 20% is none • 9% is within a day • 71% is more than a day • (8 units > 253 days).
Number of failures	Less than 8 times	• 0–9 times • 31% is none • 69% is more than 1 time	• 0–26 times • 20% is none • 80% is more than 1 time • (26 units > 9 times)
Backup and alternative unit	Yes	No	No
Maintenance Cost	Less than MYR8,000	• MYR0—MYR10,000 • 80% is none • 13% is within MYR1—MYR1,000 • 7% is within MYR1,000—MYR10,000.	• MYR0—MYR86,000 • 48% is none • 22% is within MYR1—MYR1,000 • 30% is within MYR1,000—MYR86,000.
Function (type)	• 50% (1) • 50% (3).	• 14% (1, 2) • 31% (3) • 55% (4, 5).	• 91% (1, 2) • 8% (3) • 1% (4, 5).

*The percentages stated in the table reflect to the unit of equipment*.

The medium priority of equipment corrective maintenance revealed that the highest time taken to respond to any occurring breakdown is 69 days, in which 31% is none and 15% of equipment is more than a day. The equipment requires basic cheques and average maintenance, and the rectification work is in between 0 and 253 days. No backup or alternative unit is available in the medium priority. Besides, the cost of rectification works is between MYR0 to MYR10,00, where the maintenance cost for 7% of the equipment is more than MYR1,000. For high priority, the highest reading for response time is 148 days, the repair time is 478 days, whereas the failure frequency is 29 times. The equipment requiring extensive maintenance work is equipment under high priority. No backup or alternative unit is available. Moreover, the repair work cost is in between MYR0 to MYR86,000, in which 30% of the units is more than MYR1,000.

In the high priority, medical equipment has high characteristics. Furthermore, the equipment with low and average characteristics are typical in medium priority. The only limited observation was observed with low priority equipment as only two units were involved. This is due to the less sample of equipment in low priority level. Thus, more sample of malfunctioning medical equipment is needed to obtain a clear segregation in low priority. However, as shown in Table 8, the significant indicators reasonable for a low priority are repair time, number of failures, and maintenance cost.

Referring to the Table 8, the results show that the k-Means technique measures the dataset of medical equipment without prioritising to the function or type of equipment. All the criteria contributed significantly to assess the medical equipment for corrective maintenance prioritisation. The equipment prioritisation for corrective maintenance is assigned by measuring the pattern of the nine features in the dataset, and locating the nearest Euclidean distance with the centroids. Hence, the non-critical devices in terms type and functionality can be prioritised at high corrective maintenance priority if the equipment is outdated, maintained improperly, and failed to operated frequently.

#### Replacement Programme

In the replacement programme prioritisation system, 11 medical equipment features were proposed. The output of this system was divided into three priority levels, and the quantities of medical equipment at each level are tabulated in Table 6. The clustering analysis of the replacement programme demonstrated that the low priority medical equipment consists of active units with the majority of 96% below ten (10) years as tabulated in Table 9. The manufacturer still provided the support service, and the service provider required only one time of PPM frequency, while the routine inspection was annually conducted. Moreover, the number of breakdowns was in between 0 to 11 times with the total repair work costs in between MYR0 to MYR22,000.

**Table 9:** Findings of replacement programme prioritisation system.

**Feature**	**Range**		
	**Low**	**Medium**	**High**
Age	• 0–10 years • 74% is <5 years; • 26% is in between 5 and 10 years.	• 3–30 years • 18% is <10 years • 82% is more than 10 years.	• 2–41 years; • 12% is <10 years • 88% is in between 10 and 41 years (2 units > 30 days).
Obsolescence	Available (99.6%)	Available (17%) and not available (83%)	Not available (94.2%)
Maintenance requirement	1–2 (1 × PPM frequency and routine inspection)	2–4 (1 × PPM frequency, calibration, and 2 × PPM)	3–5 (1 × PPM frequency, calibration, 2 × PPM, and statutory certification)
Number of failures	• 0–11 times • 70% is none • 30% is in between 1 and 11 times.	• 0–26 times • 27% is none • 72% is in between 1 and 11 times • 1% is in between 12 and 26 times.	• 0–41 times; • 31% is none • 64% is in between 1 and 11 times • 6% is in between 12 and 41 times (2 units > 26 times).
Maintenance Cost	• MYR0—MYR22,000 • 83% is none • 14% is within MYR1—MYR1,000 • 3% is within MYR1,000 – MYR10,000.	• MYR0—MYR86,000 • 68% is none • 17% is within MYR1—MYR1,000 • 15% is within MYR1,000—MYR86,000	• MYR0—MYR212,000 • 51% is none • 21% is within MYR1—MYR1,000 • 27% is within MYR1,000—MYR212,000 (8 units > MYR86,000).
Asset Condition	Active	Proposed for disposal and BER	Active and Proposed for disposal
Function (type)	• 35% (1, 2) • 16% (3) • 49% (4, 5).	• 41% (1, 2) • 23% (3) • 36% (4, 5).	• 55% (1, 2) • 14% (3) • 31% (4, 5).

*PPM, Planned Preventive Maintenance; BER, Beyond Economical Repair*.

*The percentages stated in the table reflect to the unit of equipment*.

The medium priority equipment comprises the equipment utilised between 3 and 30 years and require at least one time of PPM frequency and calibration. Few support services were available, but mostly no longer provided by the manufacturer or service provider. The number of failures was in between 0 and 26 times, while 15% of total equipment maintenance costs is more than MYR1,000. The equipment disposal was suggested, which required further assessment for approval, and the equipment was under the status of BER.

The characteristics of equipment categorised under high priority for replacement programme consists of 0–41 years in service and already obsolescent equipment. The equipment requires at least one time of PPM and calibration per annum and the renewal of statutory certification from the authority body. The equipment had the highest number of failures with 41 times, and the accumulative maintenance costs of MYR212,000 throughout its service life. The equipment is still active and required a quick action course by the clinical engineers for further investigation. The findings of the clustering technique on medical equipment for the replacement programme prioritisation system are presented in Table 9. The table shows that the characteristics' features indicate the segregation of the results of the cluster.

The k-Means clustering technique with 11 specified features from various categories of medical equipment effectively divided the 13,352 units into three priority levels. Similar to the results produced in preventive and corrective maintenance prioritisation systems, the k-Means clustering technique with the support of 11 features from various medical equipment categories can create a useful assessment system for the replacement programme prioritisation by selecting the distance metric of Squared Euclidean and five (5) replicate number during the cluster analysis process. Moreover, the results show that the proposed clustering technique measures the dataset of medical equipment without prioritising to the function or type of equipment. All the criteria contributed significantly in assessing the medical equipment for replacement programme prioritisation. The medical equipment is prioritised by measuring the pattern of the 11 features in the dataset, and locating the nearest Euclidean distance with the centroids. Thus, the division of clusters is not prioritised based on the specific type or functionality of the medical devices.

### Prioritisation Predictive System

#### Preventive Maintenance

The clusters segregated from the previous stage (prioritisation analysis stage) were then fed into supervised machine learning algorithms to develop a robust predictive system. The predictive system was tested on six algorithms by considering the nine features of various medical equipment categories. Several quantitative measurements were performed to evaluate the prediction performance, and the results are tabulated in Table 10. The Support Vector Machine is observed to be the best algorithm in predicting the priority of medical equipment preventive maintenance. The results also proved that this algorithm produced the highest accuracy, precision, recall, and f-score and predicted the correct classification of 13,275 medical equipment prioritisations by executing the performance evaluation.

**Table 10:** Preventive maintenance performance evaluation.

**Algorithm**	**Accuracy**	**Precision**	**Recall**	**F-score**	**Misclassification**
Decision tree	98.05%	97.83%	97.75%	97.79%	260
Linear discriminant	94.35%	94.61%	92.90%	93.75%	755
Naïve bayes	91.93%	91.86%	90.78%	91.32%	1,077
**Support vector machine**	**99.42%**	**99.38%**	**99.36%**	**99.36%**	**77**
K-Nearest neighbour	99.09%	99.02%	98.95%	98.98%	121
Bagged trees	98.87%	98.73%	98.68%	98.70%	151

Besides, the comparison of the performance evaluations tabulated in Table 10 shows that the Support Vector Machine algorithm produced a minimal misclassification of 77. Additionally, K-Nearest Neighbour seems to predict the medical equipment preventive maintenance as the parameters of performance evaluation results exceed 98.9%. The Naïve Bayes algorithm achieved the lowest performance compared to the other five classifiers.

#### Corrective Maintenance

The outputs of the corrective maintenance prioritisation system in the first stage were used as the inputs of the corrective maintenance prioritisation predictive system in the second stage. These outputs consisted of 1,028 pieces of medical equipment, and each unit was labelled with a priority number. Firstly, all eleven features were applied for models' training, and the predicted outputs were observed. The results of prediction were generated by applying the performance evaluation as tabulated in Table 11. The results showed that the K-Nearest Neighbour is the best algorithm in predicting medical equipment corrective maintenance priority. The results also proved that this algorithm produced the highest reading accuracy, precision, recall, and f-score and predicted the accurate classification of 1,017 medical equipment prioritisations by executing the performance evaluation.

**Table 11:** Corrective maintenance performance evaluation.

**Algorithm**	**Accuracy**	**Precision**	**Recall**	**F-score**	**Misclassification**
Decision tree	97.18%	64.65%	64.72%	64.69%	29
Linear discriminant	95.91%	65.28%	63.11%	64.18%	42
Naïve bayes	73.64%	55.95%	83.37%	66.96%	271
Support vector machine	98.74%	99.18%	99.00%	99.09%	13
**K-Nearest neighbour**	**98.93%**	**99.32%**	**99.14%**	**99.23%**	**11**
Bagged trees	98.05%	65.38%	65.26%	65.32%	20

The comparative analysis was conducted on the performance evaluation results as tabulated in Table 11. The analysis demonstrated that the Support Vector Machine can also be applicable for predicting the medical equipment corrective maintenance as the parameters for performance evaluation results exceed 98.7% with a minimal misclassification of 13. Decision Trees, Linear Discriminant, and Bagged Trees appear inappropriate due to lower precision, recall, and f-score, although the misclassification value was relatively low. This situation is due to inaccurate prediction of actual classification in the low priority region as the number of equipment is minimal compared to the total equipment sample.

#### Replacement Programme

The inputs of this stage were the database of labelled 13,352 medical equipment with priority numbers. The prediction results by computing the performance evaluation of all 11 features of 13,352 units were generated as tabulated in Table 12. The performance evaluation results indicated that the Support Vector Machine is the best algorithm in predicting the replacement programme priority. The performance evaluation results also proved that the algorithm produced the highest accuracy, precision, recall, f-score, and false predicting classification of 27 medical equipment prioritisations.

**Table 12:** Replacement programme performance evaluation.

**Algorithm**	**Accuracy**	**Precision**	**Recall**	**F-score**	**Misclassification**
Decision tree	99.40%	99.49%	99.53%	99.51%	80
Linear discriminant	97.90%	98.73%	97.93%	98.33%	281
Naïve bayes	98.15%	98.69%	98.12%	98.40%	247
**Support vector machine**	**99.80%**	**99.84%**	**99.81%**	**99.83%**	**27**
K-Nearest neighbour	99.66%	99.74%	99.69%	99.71%	46
Bagged trees	99.70%	99.78%	99.75%	99.76%	40

The comparison of performance evaluations as tabulated in Table 12 also shows that the classification performance of Decision Tree, K-Nearest Neighbour, and Bagged Trees also attained comparable results with a score of 99.4% of all performance evaluation parameters. The Naïve Bayes and Linear Discriminant algorithms achieved the lowest performance compared to the other four classifiers with the misclassification over 240. Generally, the performance evaluation results for all classifiers of all features and feature selection were relatively consistent.

## Discussion

### Prioritisation Assessment System

The clustering algorithm for specified features of various categories of medical equipment can develop a feasible prioritisation assessment system. The output generated can be an indicator to clinical engineers responsible for the maintenance management, execution, and supervision of medical equipment reliability to prioritise the workload in healthcare facilities. The study in the first stage of prioritisation analysis demonstrated that the comprehensive strategic maintenance management for medical equipment was successfully developed. This comprehensive strategic maintenance management covers three management exercises during the maintenance phase of the medical equipment life cycle, namely preventive maintenance, corrective maintenance, and replacement programme. None of the previous studies contributed and concentrated on comprehensive strategic maintenance management that included all these three elements.

Moreover, the analysis of clustering in identifying the strategic maintenance management priority level solely depended on the medical equipment database without users' manual intervention, which entailed vast experience and broad knowledge. The requirements of experience and knowledge are in the operation of medical equipment and implementation of maintenance management. Besides, this intervention leads to inaccuracy and subjectivity in the analysis processes (55). The priority level was analysed by measuring the medical equipment patterns and trends for the past 5 years. Therefore, the study outcomes complement the technical, clinical, and management elements of the medical equipment. Besides, the priority level outputs can be quickly generated, and consistent analysis can be produced.

The analysis demonstrates that the application of unsupervised machine learning technique able to measure the dataset of medical equipment without giving priority specifically to the function or type of equipment. All the proposed criteria contributed significantly to assess the medical equipment for the prioritisation of preventive maintenance, corrective maintenance, and replacement programme. The prioritisation is assigned by measuring the pattern of the features in the dataset and locating the nearest Euclidean distance with the centroids for every activity. Thus, the division of clusters for preventive maintenance, corrective maintenance, and replacement programme is not particularly prioritised based on the specific type or functionality of the medical devices.

The strategic maintenance management priority levels could assist the clinical engineers in understanding the possible equipment conditions and characteristics. Reporting on equipment performance can be done more comprehensively by understanding the characteristics of medical equipment. The technical report that consists of preventive maintenance, corrective maintenance, and replacement programme is significantly crucial for healthcare management in directing, preparing, and scheduling the clinical workforce to upkeep the healthcare services quality within the facility. A better understanding of prioritisation management can be achieved from the proposed predictive model. The findings are in agreement with Curtis et al. (56) who reported that convincing other professions in the healthcare sector regarding medical equipment prioritisation maintenance are challenging. However, the authors established that these challenges could be mitigated by communicating in a structured approach manner. Therefore, more structured planning can be attained by having the quantitative analysis of prioritisation analysis.

The system can be used as a supporting tool in managing the maintenance activities of medical equipment. The clinical engineers can manage financial matters appropriately by prioritising the maintenance expenses plan, such as purchasing consumable replacement parts, the internal appointment of highly skilled personnel, labour costs, materials, and tools to undertake the maintenance according to the priority level during preventive and corrective maintenance activities. The replacement plans expenditure can also be structured by prioritising high priority medical equipment to ensure the medical equipment availability is consistent to avoid healthcare service interruption. Therefore, the initial and operating expenses suggested by WHO can be structured accordingly to maintain the medical equipment in the healthcare facility (13).

Moreover, clinical engineers can manage the routine and daily activities by preparing the work schedule of preventive and corrective maintenance based on the priority levels. The indications generated from the priority systems could trigger the clinical engineers to carry out a detailed inspection to determine the actual condition of the medical equipment and the cause of failure. Proper maintenance can be performed based on the present condition. According to Kutor et al. (11), the equipment breakdown can be due to maintenance deficiency and wear-out failure. Therefore, clinical engineers can strategise a better maintenance plan to ensure the medical equipment are reliable and safe to be used by clinicians.

### Prioritisation Predictive System

The results of all classifier performance evaluations demonstrated that the Support Vector Machine and K-Nearest Neighbour are two algorithms that produce the highest prediction performance in measuring this type of dataset for all three maintenance activities. The Bagged Decision Trees performance works quite well except for corrective maintenance for all parameters where the evaluation results of precision, recall, and f-score were slightly lower. However, the Bagged Decision Trees still produced high accuracy by misclassification of 1.9%. The analysis and obtained results demonstrated that the higher prediction rates of strategic maintenance management depend on classification algorithms and the combination of proposed features from various categories of medical equipment utilised in healthcare facilities.

These proposed techniques function well in predicting the medical equipment maintenance activities and have been successfully performed in previous related studies (32, 33). The technique is also a common technique used in measuring actual data with high complexity in the healthcare sector (57, 58). Additionally, machine learning is a potential method in improving various functions in the healthcare sector. The application also enhances the diagnostic accuracy for further exploration and remedial procedures and subsequently mitigates hospital readmission, increasing service expenses (59–61).

The study findings in the second and third stages verified that the predictive prioritisation system of comprehensive strategic maintenance management was successfully established. The prediction outputs of preventive maintenance, corrective maintenance, and replacement programme performed well with high-performance evaluation results. Thus, the predictive prioritisation system may provide comprehensive prediction analyses of strategic maintenance management in healthcare facilities. The strategic maintenance management through a predictive prioritisation system may work automatically without considering the user's intervention manually. The predictive system can rapidly generate the outputs by measuring the existing medical equipment dataset simultaneously with the new dataset for quick decision-making assistance.

Furthermore, this system turns to be a robust classification approach that can work with various types of medical equipment and is not concentrated on specific equipment. The system may provide a sufficient framework in healthcare services where myriad equipment, either critical or non-critical devices, are involved. Therefore, this predictive prioritisation system can be practically implemented in any healthcare facility setup comprising hospitals, clinics, and tertiary healthcare institutions with various medical equipment applications. Besides, the system can provide a comprehensive projection of the strategic maintenance management, which offers an optimum healthcare service to prioritise the best decision-making plans.

Prediction by using machine learning classification can support clinical engineers in managing the maintenance activities, especially for a new set of medical equipment databases. The prediction process can be originated by utilising the prioritisation system outputs from the existing database, where each piece of equipment is labelled with a priority level. The machine learning tool can predict the new set of equipment outputs by measuring existing labelled data. The new prediction output can be included in the existing labelled data to better predict outcomes in future predicting processes. The clinical engineers can prepare a forecast of upcoming maintenance expenses and request for the budget in the earlier stage from this prediction outcome especially involves in replacement unit.

Moreover, the clinical engineers could strategise the best maintenance practises in light of the prediction results of preventive maintenance, corrective maintenance, and replacement programme. Preparation of early schedules for future activities would be beneficial for better implementation of maintenance. According to Belhouideg (62), insufficient use of medical equipment is one of the factors that leads to high mortality rates due to pandemics. Therefore, proper maintenance with suitable scheduling can increase the medical equipment reliability and availability in healthcare facilities. The system applies a specific method of equipment condition assessment (63) that can improve scheduling, operating stability, functional reliability, resource consumption, and spare part management (64).

Shrama et al. (65) stated that the Coronavirus (COVID-19) infected cases and mortality rate are increasing daily. This pandemic affected economic and health problems, posing a critical challenge to the entire world (66, 67). The virus flows throughout the nasal canal and travels to the human lungs within a short period of time (68). Thus, critical medical equipment utilisation such as ventilators are highly required (69, 70). Medical equipment availability and reliability must be up to the required level to provide the best healthcare services throughout this critical period. The necessity requires a fast course of action to ensure that the medical equipment can operate as the manufacturer specifications and encounter any challenges (71). Hence, the comprehensive prioritisation predictive system can provide quick assistance and indication during decision-making in prioritising the execution of preventive maintenance, corrective maintenance, and replacement programme.

### Overall Findings

The combination of 16 novel features, which are (1) age, (2) function, (3) PM status, (4) missed PPM, (5) maintenance requirement, (6) maintenance complexity, (7) downtime, (8) operations, (9) number of failures, (10) response time, (11) repair time, (12) backup and alternative unit, (13) maintenance cost, (14) asset status, (15) obsolescence, and (16) asset condition was used for prioritisation assessment and prediction of medical equipment's comprehensive strategic maintenance management. The system development measured 13,352 various types of medical equipment used in Malaysian public health clinic facilities.

The prioritisation assessment and predictive prioritisation model were developed by using the combination of unsupervised and supervised machine learning. The clustering technique of k-Means was applied in all three prioritisation assessments for preventive maintenance, corrective maintenance, and replacement programme. This technique successfully segregated the medical equipment into three priority levels which known as low, medium, and high. The segregation to appropriate priority level was based on current condition and characteristic of the equipment. Subsequently, the outputs of prioritisation assessment were used to train the predictive model by applying six classifiers which are Decision Tree, Linear Discriminant, Naïve Bayes, Support Vector Machine, K-Nearest Neighbour and Bagged Trees. The selection of the best classifiers for each activity was determined by applying five performance evaluation parameters and the comparative analysis was done among the classifiers. This combination techniques can be used as a mechanism in executing the predictive maintenance.

According to Endrenyi et al. (64), the predictive maintenance may improve the outage scheduling, operational stability, equipment reliability, resources usage, and effective spare parts management. The authors also added that the predictive maintenance is a proactive measure, which intentionally applies the specific techniques to prevent the possibility of equipment failures. Furthermore, the element of maintenance program called Reliability-Centred Maintenance is also included in predictive maintenance exercise. This is also supported by Pintelon et al. (72) stated that the predictive maintenance is more advanced than other maintenance approaches as it relies on specific inspection, state, and risk-based technique. Applying this maintenance strategy is more accessible and economy for condition monitoring equipment. Therefore, the comprehensive strategic maintenance management in this study can be used as a tool in predictive maintenance execution in optimising the reliability, availability, and safety of medical equipment.

The new set of medical equipment databases will improve the prioritisation assessment and predictive model results. This improvement allows the system to produce better accuracy and precision, and minimise the misclassification rate of prediction. Thus, by applying the techniques consistently using a greater number of medical equipment in future, the system will generate better prioritisation assessment and prediction results.

The findings in this study also demonstrated that the developed system can be replicated on various types of medical equipment used in healthcare facilities. The developed system can be a supportive tool to assist clinical engineers in overseeing the entire maintenance programme, including controlling expenditures, outlining the correct maintenance schedule, handling better resources in technical personnel and materials, preparing informative reporting documentation, and organising the replacement plan. Furthermore, it may assist the policymaker in preparing a new procedure and updating the current guidelines for more structured maintenance and procurement through early indication of predictive model. Hence, the clinical engineers can administer the management of medical equipment maintenance to ensure reliability, availability, and safety are achieved at the expected level. Consistency in terms of reliability, availability, and safety is very crucial for medical equipment especially during pandemic outbreak like COVID-19.

## Conclusions

This study demonstrated the successful development model of prioritisation assessment and predictive system by applying the unsupervised and supervised machine learning techniques, respectively. The developed model turned to be a comprehensive strategic maintenance management covering three main activities during maintenance phase of asset life cycle, which are preventive maintenance, corrective maintenance, and replacement programme. The developed model measured a dataset of medical equipment, which encompasses the combination of 16 novel features.

The prioritisation systems were developed by modifying the k-Means clustering method and this technique seems appropriate to the combination of specified features. From the performance evaluation and comparative analysis carried out on the predictive model, it can be concluded that:

- The Support Vector Machine is the best classifier for the preventive maintenance with the highest prediction accuracy of 99.42%;
- The K-Nearest Neighbour is the best classifier for the corrective maintenance with the highest prediction accuracy of 98.93%; and
- Support Vector Machine is the best classifier for the replacement programme with the highest prediction accuracy of 99.80%.

The proposed model can overcome the existing approach that requires user's manual involvement with extensive experience and broad knowledge in clinical, technical, and management elements. This study demonstrated that the developed model is robust in asssesing and predicting various types of ME utilised in any setup of healthcare facilities.

The development of a prioritisation assessment system and predictive prioritisation system utilised the historical data of medical equipment in this study. Integrating a real-time database in the asset management system in healthcare institutions will be more realistic to make both systems more practical. Integration with a real-time database will determine how the system can respond and react quickly on a regular basis to medical equipment data registration. The recommendation for further investigation on the study practicality on a real-time database will lead to the effectiveness of both systems. In addition, the benefits of integration will turn the medical equipment real-time database to be a robust and comprehensive asset management system.

## Data Availability Statement

The data used to support the findings of this study are available from the corresponding author upon request.

## Author Contributions

All authors have participated significantly to the work's conception and design, as well as the acquisition, analysis, or interpretation of data for the work, substantially to the work's authoring, critical revision for crucial knowledgeable content, approved the final version to be submitted for publication, and concurred to be accountable for all work aspects.

## Funding

This work was supported by the University Malaya Research Grant Faculty Programme (RF010-2018A) and International Funding from Motorola Solution Foundation (IF014-2019).

## Conflict of Interest

The authors declare that the research was conducted in the absence of any commercial or financial relationships that could be construed as a potential conflict of interest.

## Publisher's Note

All claims expressed in this article are solely those of the authors and do not necessarily represent those of their affiliated organizations, or those of the publisher, the editors and the reviewers. Any product that may be evaluated in this article, or claim that may be made by its manufacturer, is not guaranteed or endorsed by the publisher.
